# The FP4026 Research Database on the fundamental period of RC infilled frame structures

**DOI:** 10.1016/j.dib.2016.10.002

**Published:** 2016-10-13

**Authors:** Panagiotis G. Asteris

**Affiliations:** Computational Mechanics Laboratory, Department of Civil Engineering, School of Pedagogical & Technological Education, Athens, Greece

**Keywords:** Infilled frames, Fundamental period, Structural engineering, Masonry structures

## Abstract

The fundamental period of vibration appears to be one of the most critical parameters for the seismic design of buildings because it strongly affects the destructive impact of the seismic forces. In this article, important research data (entitled FP4026 Research Database (Fundamental Period-4026 cases of infilled frames) based on a detailed and in-depth analytical research on the fundamental period of reinforced concrete structures is presented. In particular, the values of the fundamental period which have been analytically determined are presented, taking into account the majority of the involved parameters. This database can be extremely valuable for the development of new code proposals for the estimation of the fundamental period of reinforced concrete structures fully or partially infilled with masonry walls.

**Specifications Table**

**Table:** 

Subject area	*Civil engineering*
More specific subject area	*Earthquake engineering*
Type of data	*Table, figure*
How data was acquired	*Survey*
Data format	*Raw, analyzed*
Experimental factors	The frames are designed according to Eurocode standards prior to analysis
Experimental features	All frames were modelled using Seismostruct [4]
Data source location	*n/a*
Data accessibility	*Data is with this article*

**Value of the data**

• The FP4026 Research Database is associated with the values of the fundamental period of vibration of RC masonry infilled frame structures up to 24 floors.
• The research data is important for researchers who deal with earthquake design of structures.
• The data can be extremely valuable for the development of new code proposals for the estimation of the fundamental period

## 1 Data

Here we present the main characteristics of the FP4026 Research Database (Fundamental Period-4026 cases of infilled frames), which comprises values of the fundamental period of masonry infilled reinforced concrete framed structures.

The FP4026 Research Database presents the values of the fundamental period of a large set of infilled frames that have been analytically estimated, taking into account the majority of the involved geometrical and mechanical parameters such as the number of storeys, the number of spans, the span length, the percentage of the opening in infill was and the stiffness of the infill wall panels.

## 2 Experimental design, materials and methods

**Fig. 1:**
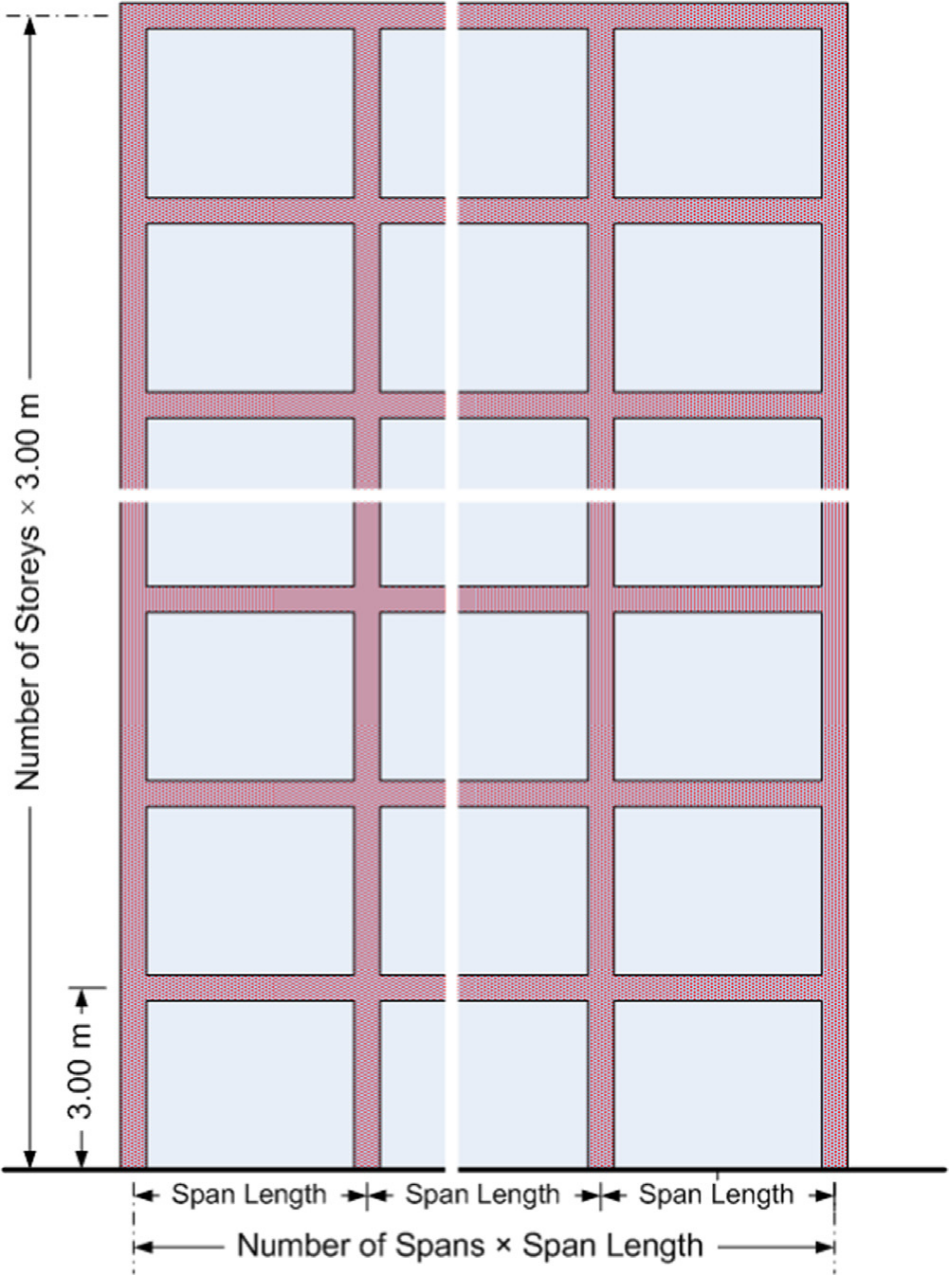
Cross section details of an infilled RC frame.

A total of 4026 infilled plane reinforced concrete frames have been investigated; the quantitative outcomes of these cases are included in the FP4026 Research Database. Specifically, the number of storeys was ranged from 1 to 22, investigated by upgrading the number of storeys by unit increments (Fig. 1). The storey height for all buildings is kept constant and equal to 3.0 m. The number of spans varied between 2, 4 and 6. For each case, four different span lengths were considered, namely 3.0 m, 4.5 m, 6.0 m and 7.5 m. In the perpendicular direction the span length has been kept constant and equal to 5 m for all cases.

Bare frame structures as well as structures with fully or partially unreinforced masonry infilled frames with or without openings have been analysed, in order to examine the influence of infill walls. A lot of parameters have been considered for each case. Infill panels are either 0.15 or 0.25 m thick, following the conventional construction of single and double leaf walls. The influence of infill wall openings is also examined. Infill wall openings are given as a percentage of the panel area. Five different cases for infill wall openings are studied. These are: 0% openings (fully infilled masonry panels), 25%, 50%, 75% and 100% openings (case of bare frames –no openings such as windows and doors). Moreover, five different values for the masonry panel strength were adopted to represent weak, medium and strong masonry, namely 1.5 MPa, 3.0 MPa, 4.5 MPa, 8.0 MPa and 10.0 MPa. Detail and in-depth description for each one of masonry panel strength is presented in the supplementary document. Specifically, in the sheet of the MS-Excel file under the title “Masonry Wall Stiffness” the values of the masonry panel strength have been presented.

The building parameters used for the development of the model are listed in Table 1. In total, 4026 different cases of infilled RC frames were analysed in order to investigate the influence of several parameters on the fundamental period of a frame structure.

The frames are designed according to the Eurocode 8 standards [1], [2], [3]. Modal response spectrum analysis has also been performed. The frames were designed for seismic zone I with reference peak ground acceleration on type A ground, agR=0.16 g, importance factor *γI* equal to 1.0, ground type B with soil factor *S* equal to 1.2, according to Eurocode 8, medium ductility class (DCM) and behaviour factor, *q*, equal to 3.45. Concrete strength class C25/30 was used for beams and columns, while steel grade B500c was used for the reinforcement steel bars. The dead load was 1.50 kN/m^2^ plus 0.90 kN/m^2^ to include interior partition walls in the mass of the building. Live load was 3.5 kN/m^2^. Slabs were 150 mm thick for all cases. Beams were 250/600 mm for all frames. Square column sections were used for all frames. Detail and in-depth description for each one square column sections is presented in the supplementary document. Specifically, in the sheet of the MS-Excel file under the title “Columns Sections” the dimensions of the column sections have been presented. Column longitudinal reinforcement ratio was kept low and ranged between 1.0% and 1.5%, with most cases being under 1.15%.

**Fig. 2:**
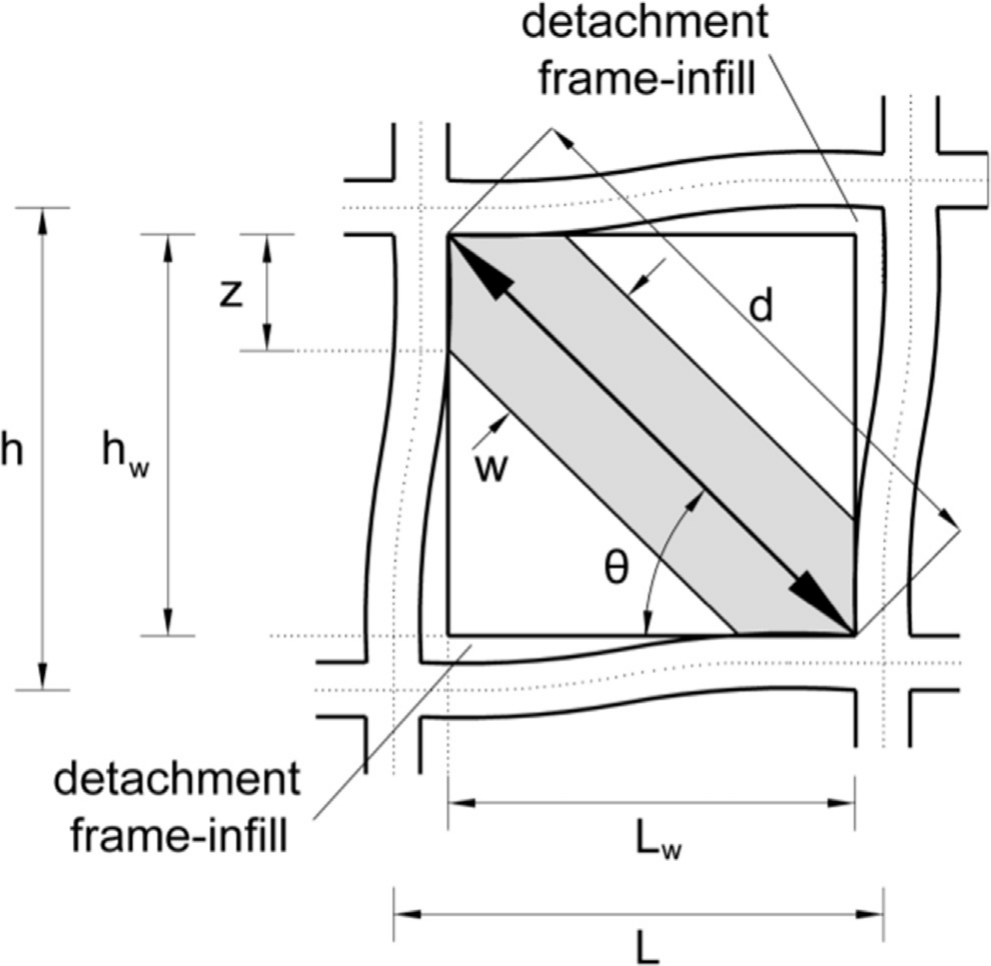
Masonry infill frame sub-assemblage.

Since the first attempts to model the response of the composite infilled-frame structures, experimental and conceptual observations have indicated that a diagonal strut with appropriate geometrical and mechanical characteristics could possibly provide a solution to the problem (Fig. 2). In Fig. 2 *w* is the width of the diagonal strut, *d* is the diagonal length of the masonry panel, *L* is the distance between the centres of two columns and *z* is the contact length of the diagonal strut to the column.

All buildings were modelled as plane frames using Seismostruct [4]; masonry infill walls have been modelled using an equivalent strut nonlinear cyclic model proposed by Crisafulli [5]. Asteris [6], [7], [8], [9], [10], [11] proposed a finite element technique in order to estimate the infill/frame contact lengths and this technique has since been used to investigate the effect of openings on the lateral stiffness of masonry infill walls. Based on these findings the following relationship for the infill wall stiffness reduction factor *λ* had been proposed and implemented:

(1) $$\lambda =1-{2\alpha }_{W}^{0.54}{+\alpha }_{W}^{1.14}$$

where $\alpha_{W}$ is the ratio of the area of opening to the area of infill wall.

**Fig. 3:**
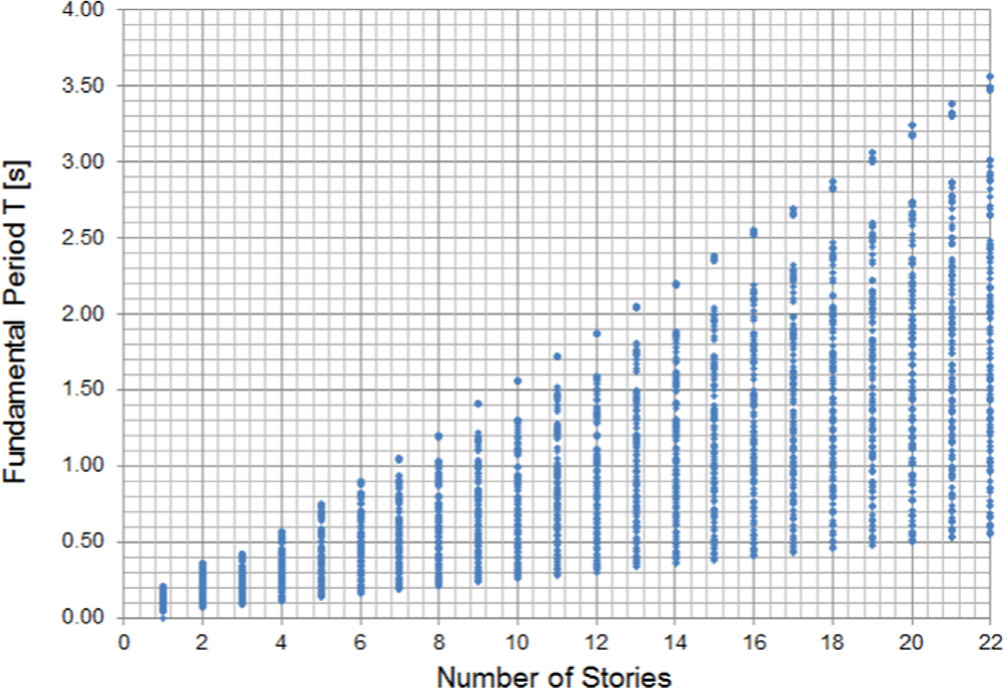
Fundamental period vs number of stories of RC frame structures.

The values of the fundamental period of all cases of infilled frames studied are presented in Fig. 3 as well as in the sheet of the MS-Excel file under the title “Fundamental Periods” in the supplementary document.

## Figures and Tables

**Table 1:** Building parameters.

Concrete strength	25.00 MPa
Modulus of elasticity of concrete, *E*_*c*_	31.00 GPa
Steel tensile yield strength	500.00 MPa
Size of beams	250/600 mm
Slab thickness	150 mm
Dead loads	1.50 kN/m^2^ + 0.90 kN/m^2^
Live loads	3.50 kN/m^2^
Number of floors	1 to 22 by 1
Storey height	3.00 m
Span length	3.00 m, 4.50 m, 6.00 m, 7.50 m
Number of spans	2, 4, 6
Masonry compressive strength, *f_m_*	1.50 MPa, 3.00 MPa, 4.50 MPa, 8.00 MPa, 10.0 MPa
Modulus of elasticity of masonry, *E*_*m*_	1.50 GPa, 3.00 GPa, 4.50 GPa, 8.00 GPa, 10.00 GPa
Thickness of infill panel, *t*_*w*_	150 mm, 250 mm
Infill wall opening percentage	0% (fully infilled), 25%, 50%, 75%, 100% (bare frame)
